# Roseoside Is a Bioactive Compound in *Kirengeshoma koreana* Nakai Extract with Potent In Vitro Antiviral Activity Against Hepatitis C Virus

**DOI:** 10.3390/molecules29215130

**Published:** 2024-10-30

**Authors:** Jun-Kyu Lee, Ji-Wan Choi, InWha Park, Na-Eun Kim, Hak Cheol Kwon, Jaeyoung Kwon, Yoon-Jae Song

**Affiliations:** 1 Department of Life Science, Gachon University, Seongnam 13120, Republic of Korea; wnsrb0855@naver.com (J.-K.L.); chlwldhks14@naver.com (J.-W.C.); kne951027@naver.com (N.-E.K.); 2 Natural Product Informatics Research Center, Korea Institute of Science and Technology (KIST) Gangneung Institute, Gangneung 25451, Republic of Korea; inwha129@skku.edu (I.P.); hkwon@kist.re.kr (H.C.K.)

**Keywords:** hepatitis C virus, antiviral, *Kirengeshoma koreana* Nakai, roseoside

## Abstract

Hepatitis C virus (HCV) is a pathogen that causes cirrhosis and hepatocellular carcinoma through chronic hepatitis C. This study focused on the anti-HCV activity of a 70% ethanol extract of *Kirengeshoma koreana* Nakai (KKE) and its bioactive chemical constituent(s). The KKE and its *n*-butanol (*n*-BuOH) fraction induced a significant reduction in HCV RNA levels without inducing cytotoxicity. A high-performance liquid chromatography–mass spectrometry (HPLC-MS) analysis revealed the presence of roseoside in the *n*-butanol fraction of the KKE, which inhibited HCV RNA replication in a concentration- and time-dependent manner without exerting cytotoxicity. Consistent with in silico molecular docking analysis data, roseoside targets and inhibits HCV NS5A/B replicase. Collectively, our findings demonstrate that roseoside is a chemical constituent in KKE that interferes with HCV replication by targeting NS5A/B replicase.

## 1. Introduction

HCV is a major causative agent of serious liver illness, including cirrhosis and hepatocellular carcinoma and lifelong chronic hepatitis [1,2]. According to the World Health Organization (WHO), an estimated 71 million people are chronically infected by this virus and 400,000 die of HCV-related diseases on an annual basis. Furthermore, 1.75 million new HCV infections are recorded every year [3]. HCV, a member of the family *Flaviviridae*, is an enveloped, single-stranded, positive-sense RNA virus [4]. The viral genome of 9.6 kb comprises a single open reading frame (ORF) encoding a polyprotein precursor of ~3000 amino acids, which is processed by cellular and viral proteases to generate at least 10 viral proteins [5]. Structural proteins (Core, E1 and E2) are located at the N-terminus of the polyprotein, while the C-terminal regions consist of non-structural proteins (p7, NS2, NS3, NS4A, NS4B, NS5A and NS5B) [6,7]. Despite extensive research, commercialized HCV vaccines are not available as yet due to complications associated with genetic diversity [8]. Various drugs for the treatment of HCV have been developed, including sofosbuvir and velpatasvir, which are approved by the FDA. Current direct-acting antiviral agents (DAAs) for HCV operate as inhibitors of NS3/4A protease and NS5A/B RNA-dependent RNA polymerase (RdRp). However, the ongoing discovery of resistance-associated substitutions (RASs) in response to these antiviral drugs remains a significant concern [9,10,11]. For the resolution of drug resistance in the context of HCV treatment, a combination regimen incorporating drugs with diverse mechanisms of action may be effective. However, to combat the occurrence of multiple RASs, the development of novel drugs with distinct mechanisms of action from those employed previously is essential. In addition, drug treatment is expensive and inaccessible in resource-limited countries where HCV infections mainly occur [12]. These challenges highlight the necessity for rapid advancements in the research and development of novel antivirals against HCV.

Natural products derived from medicinal plants have been explored in a wide range of fields as potential sources of novel therapeutic agents. Numerous beneficial compounds and biological activities of plant extracts have been uncovered to date, leading to the ever-increasing application of medicinal plants to improve individual health or treat diseases worldwide [13,14,15]. Drugs derived from natural sources could be a suitable alternative to conventional synthetic drugs that exert side effects and provide fundamental information for new drug development. Accordingly, antiviral research in recent years has extensively focused on the therapeutic potential of various medicinal plant extracts and their derived fractions [16]. Numerous natural compounds have been shown to exert antiviral effects against HCV, such as silymarin, quercetin, curcumin, α-mangostin, piperine, β-sitosterol, oligostilbene, vaticanol B and hesperidin [17,18,19,20,21,22,23,24,25].

*Kirengeshoma koreana* Nakai (*K. koreana*) is a Korean endemic flora that was originally identified as a small population in Mt. Baekunsan of Jeolla Province [26,27]. To our knowledge, the majority of studies to date have focused on ecological and taxonomic aspects, and no information is currently available on the constituents and biological activities of K. koreana extract. The main objective of the present study was to examine the anti-HCV activity of a 70% ethanol extract of *Kirengeshoma koreana* Nakai (KKE) and identify its bioactive compound(s) with the greatest inhibitory effect on HCV replication.

## 2. Results

### 2.1. Antiviral Activities of KKE and Solvent Fractions Against HCV

**Figure 1:**
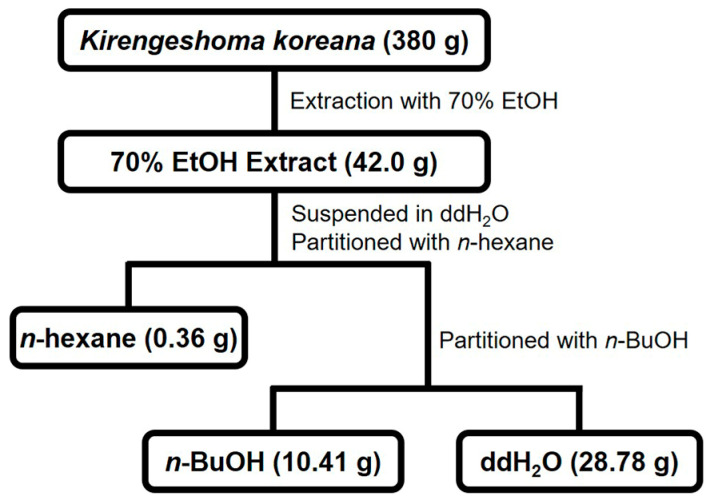
Fractionation scheme of KKE.

**Figure 2:**
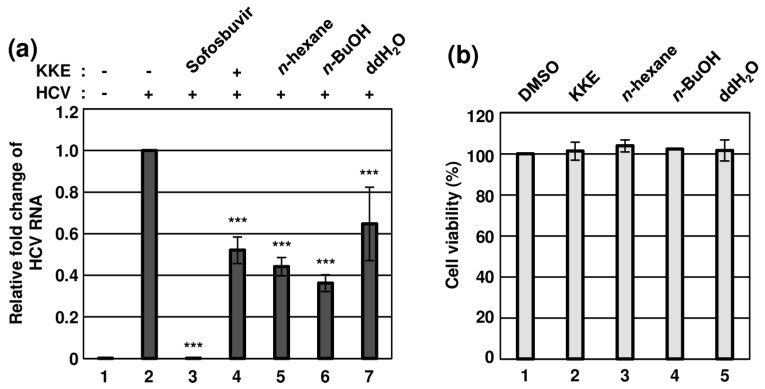
Effects of KKE solvent fractions on HCV infection and cell viability. (**a**) Naïve Huh7.5 cells were inoculated with control DMEM culture medium (lane 1) or HCVcc supernatant (lanes 2 to 7) at 0.1 MOI and treated with each sample: DMSO (vehicle control) (lanes 1 and 2), sofosbuvir (inhibitor control) at 1 μM (lane 3), KKE (lane 4), *n*−hexane (lane 5), *n*−BuOH (lane 6) or ddH_2_O (lane 7) fractions of KKE at 10 μg/mL. After inoculation, the culture medium was replaced with each sample at identical concentrations. At 48 hpi, relative cellular HCV RNA levels in the groups were measured using RT−qPCR with specific primers for HCV NS5B or GAPDH. The relative fold change of HCV RNA of the vehicle control (DMSO) group was defined as 1. (**b**) Huh7.5 cells were treated with DMSO, KKE or solvent fractions of KKE (*n*−hexane, *n*−BuOH or ddH_2_O) at a fixed concentration of 10 μg/mL, and viability was measured at 48 h post−treatment using the CellTiter−Glo luminescent Cell Viability Assay. Cell viability was determined based on relative luciferase activity, with viability of the DMSO group defined as 100%. An asterisk (*) indicates a significant difference between the control and experimental groups, which was determined by a one-way ANOVA followed by a Dunnett’s post hoc test (*** *p* < 0.001). The data are representative of three independent experiments.

*K. koreana* was dried, ground and extracted with 70% ethanol, followed by concentration as a crude extract (42.0 g) that was fractionated via sequential solvent partitions (Figure 1). The antiviral effects of KKE and its solvent fractions against HCVcc infection were evaluated. To this end, naïve Huh7.5 cells were infected with HCVcc at an MOI of 0.1 and treated with DMSO, KKE or KKE fractions. The level of HCV RNA in cells was determined via RT-qPCR with specific primers for NS5B at 48 hpi. Sofosbuvir, an inhibitor of HCV NS5B polymerase, was employed as a control. Compared to the DMSO treatment group, the HCV RNA level was significantly reduced following KKE (52%), *n*-hexane (44%), *n*-BuOH (36%), ddH_2_O (65%) and sofosbuvir (0.06%) treatment (Figure 2a). Notably, the *n*-BuOH fraction of the KKE exerted the strongest inhibitory effect against HCV infection with an 50% effective concentration (EC_50_) value of 11.63 μg/mL (Figure 2a, lane 6). To establish whether the inhibitory effects of KKE fractions on HCV infection are associated with cytotoxicity, Huh7.5 cells were treated with DMSO or solvent fractions, and cell viability was measured at 48 h post-treatment. Compared to the DMSO vehicle control, the KKE fractions did not exert measurable cytotoxic effects on Huh7.5 cells at 48 h post-treatment (Figure 2b). The 50% cytotoxicity concentration (CC_50_) of the *n*-BuOH fraction in Huh7.5 cells was estimated as 283.9 μg/mL. Thus, the selective index (SI) was calculated as 24.5. The data collectively suggest that the KKE and its fractions exhibit inhibitory activity against HCV, of which the *n*-BuOH fraction is the most effective without inducing cytotoxicity. Accordingly, further experiments were conducted to validate the antiviral efficacy of the *n*-BuOH fraction of the KKE.

### 2.2. HPLC-MS Identification of Chemical Constituents of the n-BuOH Fraction of the KKE

**Figure 3:**
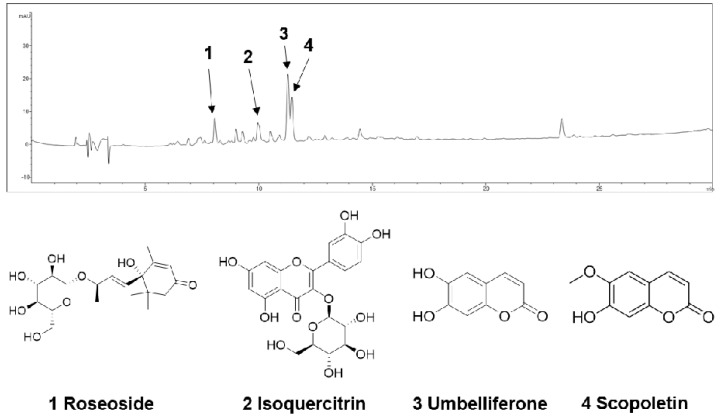
HPLC−MS analysis of the n-BuOH fraction of the KKE.

**Figure 4:**
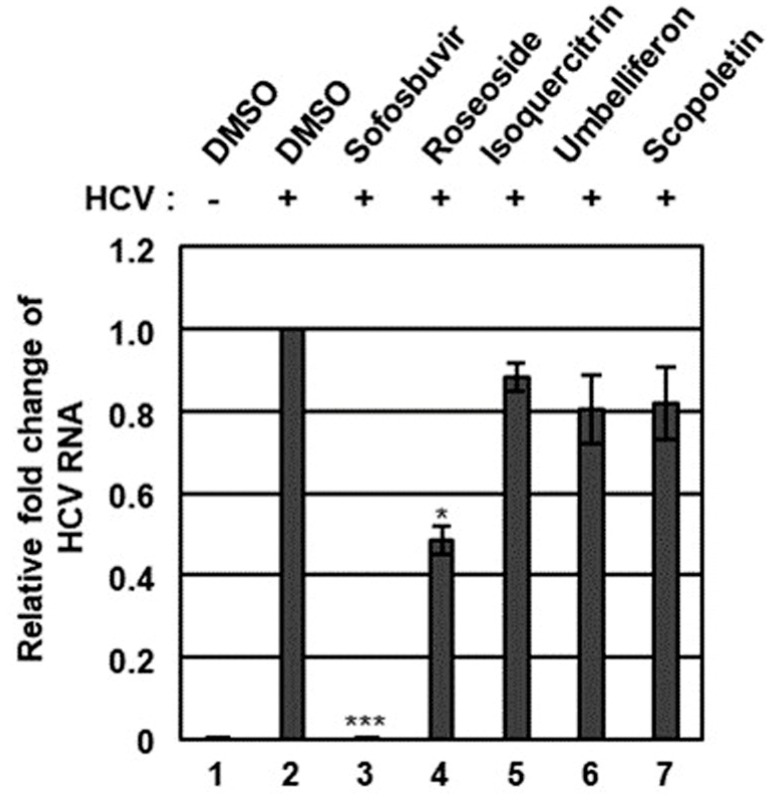
Effects of chemical constituents in the n-BuOH of the KKE on HCV infection. Naïve Huh7.5 cells were inoculated with control DMEM culture medium (lane 1) or HCVcc supernatant (lanes 2 to 7) at 0.1 MOI and treated with DMSO (vehicle control) (lanes 1 and 2), sofosbuvir (inhibitor control) at 1 μM (lane 3) and chemical constituents in the n-BuOH fraction of the KKE at 40 µM: roseoside (lane 4), isoquercitrin (lane 5), umbelliferon (lane 6) and scopoletin (lane 7). At 48 hpi, relative HCV RNA levels in cells were measured using RT-qPCR with specific primers for HCV NS5B or GAPDH. The relative fold change of HCV RNA of the vehicle control (DMSO) group was defined as 1. An asterisk (*) indicates a significant difference between the control and experimental groups, which was determined by a one-way ANOVA followed by a Dunnett’s post hoc test (* *p* < 0.05 and *** *p* < 0.001). The data are representative of three independent experiments.

The n-BuOH fraction of the KKE was further analyzed using HPLC-MS to identify the major chemical constituents (Figure 3). The major peaks in the mass and UV chromato-grams were identified as roseoside (tR 8.0), isoquercitrin (tR 10.0), umbelliferon (tR 11.4) and scopoletin (tR 11.5), with reference to previous findings from chemical databases. The antiviral effects of the chemical constituents identified in the n-BuOH fraction of the KKE against HCV were further evaluated. To this end, naïve Huh7.5 cells were infected with HCVcc at an MOI of 0.1 and treated with DMSO, roseoside, isoquercitrin, umbelliferon and scopoletin. The level of HCV RNA in cells was determined via RT-qPCR with specific primers for NS5B at 48 hpi. Compared to the DMSO treatment group, the HCV RNA level was significantly reduced following roseoside (48%) but not isoquercitrin (88%), umbelliferone (80%) and scopoletin (82%) treatment (Figure 4).

### 2.3. Concentration-Dependent Anti-HCV Activity of Roseoside with No Cytotoxicity

**Figure 5:**
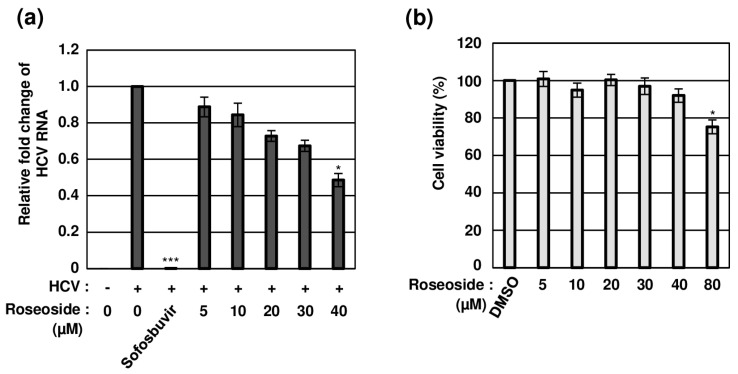
Concentration-dependent antiviral activity of roseoside against HCV with no cytotoxicity. (**a**) Naïve Huh7.5 cells were inoculated with the HCVcc supernatant (MOI of 0.1) and treated with DMSO or roseoside at a range of concentrations (5, 10, 20, 30 and 40 μM). At 48 hpi, relative HCV RNA levels in cells were measured using RT-qPCR with specific primers for HCV NS5B or GAPDH. The relative fold change of the HCV RNA of the vehicle control (DMSO) was defined as 1. (**b**) Huh7.5 cells were treated with DMSO or roseoside at a range of concentrations (5, 10, 20, 30, 40 and 80 μM), and viability was measured at 48 h post-treatment using the CellTiter-Glo luminescent Cell Viability Assay. Cell viability was determined based on relative luciferase activity, with viability of the DMSO group defined as 100%. An asterisk (*) indicates a significant difference between the control and experimental groups, which was determined by a one-way ANOVA followed by Dunnett’s post hoc test (* *p* < 0.05 and *** *p* < 0.001). The data are representative of three independent experiments.

To establish whether roseoside inhibits HCV in a concentration-dependent manner, naïve Huh7.5 cells were treated with DMSO or varying concentrations of roseoside. Compared to the DMSO-treated cells, the HCV RNA content was significantly decreased to 88%, 84%, 72%, 67% and 48% in cells treated with the roseoside at doses of 5, 10, 20, 30 and 40 μM, respectively, indicating a concentration-dependent inhibitory effect (Figure 5a). The 50% effective concentration (EC_50_) value of roseoside against HCVcc was estimated as 38.92 μM. For the examination of potential cytotoxicity, the viability of Huh7.5 cells treated with different concentrations of roseoside (5, 10, 20, 30, 40 and 80 μM) for 48 h was measured. Relative to the DMSO vehicle control, the roseoside exerted no marked cytotoxicity against Huh7.5 cells at concentrations lower than 80 μM (Figure 5b). Our data clearly demonstrate that roseoside inhibits HCV infection in a concentration-dependent manner without exerting cytotoxicity.

### 2.4. Inhibition of HCV Replication by Roseoside

**Figure 6:**
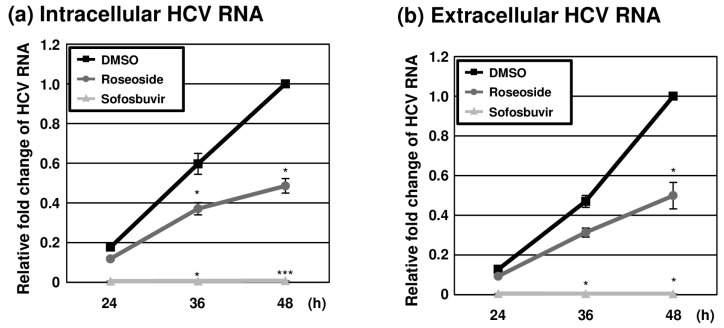
Inhibition of HCV replication by roseoside. Naïve Huh7.5 cells were inoculated with HCVcc at an MOI of 0.1 and treated with DMSO, roseoside (40 μM) or sofosbuvir (1 μM). At 4 h after inoculation, the inoculum was removed, and the culture medium was replaced with each drug. At 24, 36 and 48 hpi, HCV RNA levels in (**a**) cells and (**b**) the culture supernatantwere determined using RT-qPCR with specific primers for HCV NS5B or GAPDH. HCV RNA levels were normalized with cellular GAPDH RNA levels. The relative HCV RNA level in the DMSO-treated group at 48 hpi was established as 1. An asterisk (*) indicates a significant difference between the DMSO- and sample-treated groups at each time point, determined by a two-way ANOVA followed by a Tukey’s post hoc test (* *p* < 0.05 and *** *p* < 0.001). The data are representative of three independent experiments.

To further examine the anti-HCV activity of roseoside during the time course of infection, naïve Huh7.5 cells were infected with HCVcc (MOI of 0.1) and treated with DMSO or roseoside at a concentration of 40 μM. At different time points (24, 36 and 48 hpi), RT-qPCR analyses of the HCV RNA was performed. The HCV RNA content of the control DMSO-treated cells increased over time until 48 hpi (Figure 6a). The HCV RNA level was increased at 36 and 48 hpi compared to 24 hpi in both the DMSO- and roseoside-treated groups. Relative to the DMSO treatment group, at 24, 36 and 48 hpi, the level of HCV RNA was reduced to 67%, 62% and 49%, respectively, in the roseoside treatment group, although the decrease in HCV RNA at 24 hpi was not significant. Consistent with the observed reduction in intracellular HCV RNA, extracellular viral RNA levels were significantly decreased (Figure 6b). This continuous suppression of HCV RNA could be attributed to a time-dependent inhibition of viral replication by roseoside.

### 2.5. Roseoside Targets and Inhibits HCV NS5A/B RdRp

**Figure 7:**
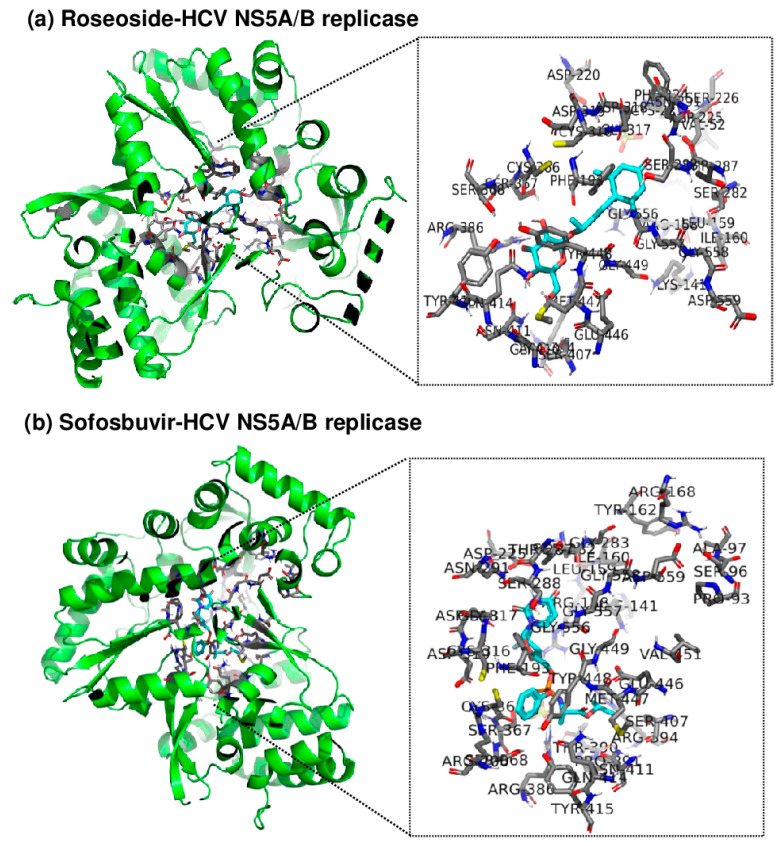
Three-dimensional visualization of the binding site between roseoside or sofosbuvir (cyan) and HCV NS5A/B replicase (green) using PyMOL. Amino acid residues at the active site of NS5A/B replicase within 4 Å of (**a**) roseoside or (**b**) sofosbuvir are highlighted, and the protein is represented in a cartoon ribbon model structure.

Since roseoside inhibits HCV replication, an in silico molecular docking analysis of roseoside at the active site of HCV NS5A/B replicase was performed using AutoDock Tools (AutoDock and AutoDock Vina) and PyMOL to uncover its mechanism of action (Figure 7). AutoDock Vina predicted the binding affinity of roseoside to HCV NS5A/B to be −7.5 kcal/mol, with both the upper and lower bounds of the root mean square deviation (RMSD u.b. and RMSD l.b., respectively) at 0, indicating the optimal binding mode. The optimal binding mode was visualized in 3D using PyMOL. By utilizing the PyMOL command line, we identified the binding of roseoside at the active site of HCV NS5A/B replicase and labeled the amino acid residues within 4 Å of roseoside. Our results illustrate the overall binding of roseoside (cyan) and the HCV NS5A/B replicase (green) using a cartoon ribbon model structure, with the binding site and surrounding amino acid residues at the enzyme’s active site highlighted in stick representation (Figure 7). The binding affinity of roseoside to HCV NS5A/B was lower than that of sofosbuvir, which is −8.7 kcal/mol. Sofosbuvir maintains structural stability by forming numerous hydrogen bonds and ionic interactions with the palm and finger domains of the active site of HCV NS5A/B replicase, whereas roseoside primarily binds through hydrophobic interactions with these domains. Sofosbuvir forms strong hydrogen bonds with residues such as Asp318, Arg386 and Thr287 within the binding site of HCV NS5A/B, thereby enhancing binding stability. In contrast, roseoside forms a hydrogen bond with Thr287 but mainly interacts hydrophobically with other residues, resulting in relatively lower binding energy compared to sofosbuvir.

**Figure 8:**
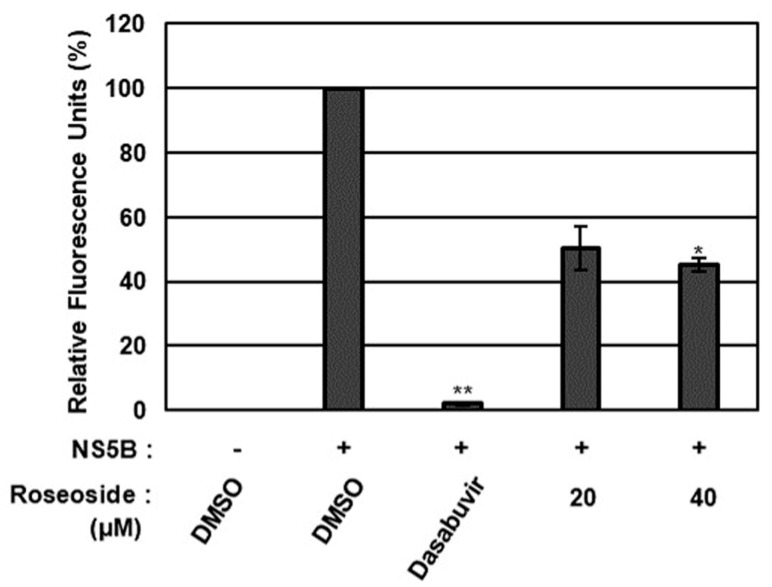
Inhibitory effect of roseoside on HCV NS5A/B activity. The activity of HCV NS5A/B replicase was determined fluorometrically using SYTO RNASelect Green Fluorescent Cell Stain after treatment with DMSO, dasabuvir (1 μM) or roseoside (20 and 40 μM). HCV NS5A/B replicase activity was expressed as relative fluorescence units with the activity in the DMSO-treated group set to 100%. An asterisk (*) indicates a significant difference between the control and experimental groups, as determined by one-way ANOVA followed by Dunnett’s post hoc test (* *p* < 0.05 and ** *p* < 0.01). The data are representative of three independent experiments.

To validate the molecular docking analysis results, the effect of roseoside on HCV NS5A/B replicase was further evaluated using an in vitro NS5A/B replicase assay (Figure 8). Compared to the DMSO-treated control, HCV NS5A/B replicase activity was significantly decreased to 50% and 45% in the presence of roseoside at 20 and 40 μM, respectively (Figure 8). Taken together, these data suggest that roseoside targets NS5A/B replicase to inhibit HCV replication.

## 3. Discussion

In this study, we evaluated the antiviral efficacy of a KKE and its chemical constituents using an HCV genotype 2a JFH-1 cell culture system. Our data showed that the *n*-BuOH fraction of the KKE contains bioactive chemical constituents that inhibit HCV replication. Roseoside, isoquercitrin, umbelliferon and scopoletin were identified as major chemical constituents of the *n*-BuOH fraction of the KKE via HPLC-MS analysis. Among the identified major chemical constituents, roseoside exhibited concentration- and time-dependent inhibitory effects against HCV RNA replication.

Roseoside is a megastigmane glycoside widely distributed in plants, and its biological activities have not been well documented [28]. It exhibits insulin mimetic activity and inhibits glucose-6-phosphatase, α-glucosidase and cyclooxygenase-2 (COX-2) activities as well as the expression of angiotensin II receptor 1 [29,30,31,32]. In addition to its insulin mimetic activity, roseoside exhibits antioxidant and anticancer activities [33,34,35]. It has been reported that roseoside inhibits Epstein-Barr virus (EBV) early antigen (EBV-EA) activation induced by 12-O-tetradecanoylphorbol-13-acetate (TPA) and shows potential binding affinity to the main protease and spike glycoprotein of severe acute respiratory syndrome coronavirus 2 (SARS-CoV-2) in in silico studies [34].

The results of the in silico molecular docking analysis suggest that roseoside has the potential to bind to the active site of HCV NS5A/B replicase, and this inhibition was verified through an in vitro NS5A/B replicase assay. The collective findings indicate that roseoside interferes with HCV replication, without cytotoxicity, by targeting NS5A/B replicase. The exact mechanism by which roseoside binds to and inhibits HCV NS5A/B replicase needs to be elucidated in future studies. Additionally, the possibility that roseoside may inhibit HCV replication by targeting cellular factors should not be overlooked.

To the best of our knowledge, this study is the first to uncover the biological activity of a KKE and its bioactive chemical constituent, roseoside, highlighting their potential value as natural sources of anti-HCV agents. In addition to the *n*-BuOH fraction of the KKE, the HCV inhibitory effect observed in the *n*-hexane fraction suggests that further studies are needed to investigate the possibility of other chemical constituents in KKEs that may interfere with HCV replication. Last but not least, further studies are needed to elucidate the impact of the KKE and its chemical constituents, including roseoside, on cellular physiology and their efficacy against other microorganisms.

## 4. Materials and Methods

### 4.1. Plant Material, Cells and Reagent

K. koreana was artificially cultivated and supplied by Hantaek Botanical Garden (Yongin-Si, Gyeonggi-Do, Korea). A voucher specimen (HTS2022-0101, HTS2022-0102) was authenticated by Dr. Jung Hwa Kang (Hantaek Botanical Garden) and deposited at Hantaek Botanical Garden. Human hepatocellular carcinoma cells (Huh 7.5) were maintained as described previously [36]. Sofosbuvir was adopted as a positive inhibitor control against HCV (MedChemExpress, Monmouth Junction, NJ, USA). Isoquercitrin, umbelliferone and scopoletin were purchased from MedChemExpress LLC (Monmouth Junction, NJ, USA). Roseoside was purchased from Biosynth AG (Gstaad, Switzerland).

### 4.2. Extraction and Partition

The aerial parts of *K. koreana* were dried for 7 days at 30 ± 2 °C in a hot air dryer. Dried *K. koreana* (380 g) was ground and extracted with 70% ethanol (6 L × 2) for 7 days at room temperature. After concentration using a rotary evaporator, the crude extract (42.0 g) was suspended in double-distilled water (ddH_2_O)(0.1 L) and partitioned with *n*-hexane (0.2 L × 3) followed by *n*-butanol (*n*-BuOH) (0.2 L × 2) to generate *n*-hexane- (0.36 g), *n*-BuOH (10.41 g), and ddH_2_O fractions (28.78 g), respectively.

### 4.3. Viruses

Genotype 2a JFH-1 strain is currently the only HCV strain with a well-established viral culture system. The genotype 2a JFH-1 strain HCV cell culture (HCVcc) was produced as described previously [37]. Briefly, a JFH-1 expression construct (provided by T. Wakita, National Institute of Infectious Diseases and Toray Industries, Tokyo, Japan) was linearized and transcribed into full-length JFH-1 RNA in vitro. Huh7.5 cells were transfected with transcribed RNA and the cell culture supernatant used for the replication of HCV. The supernatant of the HCV-infected cells was filtered through a 0.45 μm syringe filter (Sartorius, Göttingen, Germany) and used for HCV infection [38]. For the drug treatment assay, Huh7.5 cells were seeded on a 12-well plate and incubated overnight. At 90% confluence, cells were inoculated with HCVcc at a 0.1 multiplicity of infection (MOI) and further incubated for 4 h. Next, the inoculum was removed, and cells were washed twice with phosphate-buffered saline (PBS) followed by changes in the culture medium. Drug treatment was continuously maintained at all stages, from inoculation to harvesting cells and the culture supernatant.

### 4.4. Quantification of the Viral Genome

A quantitative analysis of RNA was performed using reverse transcription and real-time quantitative polymerase chain reaction (RT-qPCR) as described previously [36]. Briefly, total RNA from Huh7.5 cells or the culture supernatant was isolated using TRI reagent (Molecular Research Center, Inc., Cincinnati, OH, USA) and reverse-transcribed into cDNA using random hexamers and a TOPscript™ cDNA Synthesis kit (Enzynomics, Daejeon, Korea). An RT reaction was performed at 25 °C for 10 min, 50 °C for 1 h and 95 °C for 5 min. RT-qPCR reactions were performed using 1X HOT FIREPol EvaGreen qPCR Mix Plus (Solis BioDyne, Tartu, Estonia) in keeping with the manufacturer’s instructions. The primer sequences for the RT-qPCR were as follows: HCV NS5B, 5′-GGCTGGGAAACATCATCCAGTA-3′ (forward) and 5′-TCAAAGTTGAGGTTCTGGTCCAG-3′ (reverse); GAPDH, 5′-CATGAGAAGTATGACAACAGCCT-3′ (forward) and 5′-AGTCCTTCCACGATACCAAAGT-3′ (reverse). The thermocycling conditions for the RT-qPCR were as follows: 95 °C for 15 min followed by 40 cycles of 95 °C for 15 s, 54 °C for 20 s and 72 °C for 15 s. GAPDH was used as a reference gene for the HCV RNA. The relative amounts of HCV RNA in cells were calculated using the 2-ΔΔCT method [39].

### 4.5. Cell Viability Assay

Huh7.5 cells were seeded on 96-well cell culture plates and treated with drugs upon reaching 90% confluence. At 48 h after treatment, cell viability was evaluated with the Cell Titer-Glo^®^ Luminescent Cell Viability Assay according to the manufacturer’s instructions (Promega, Madison, WI, USA). The luminescence signal was measured using the GloMax^®^ Multi Detection System (Promega).

### 4.6. Statistical Analysis

Data are expressed as mean ± standard deviation (SD) of three independent experiments. Statistical analyses were conducted using GraphPad Prism 7 (version 7.04) (GraphPad Software, Inc. San Diego, CA, USA). A one-way analysis of variance (ANOVA) was employed to validate the statistical significance of the results with respect to variations in a single factor, such as type or concentration of drug, among more than three groups. Dunnett’s post hoc test was additionally applied for comparisons between multiple experimental treatment groups and a single control group (DMSO). A two-way ANOVA with Turkey’s post hoc test was employed for comparisons between multiple groups. An asterisk (*) indicates significant differences in the mean values between the DMSO control and drug treatment groups (*p* < 0.05).

### 4.7. High-Performance Liquid Chromatography–Mass Spectrometry (HPLC-MS) Analysis of KKE

The HPLC-MS data were recorded on an Agilent 1200 system connected to a 6120 quadrupole MSD (Santa Clara, CA, USA) equipped with a Phenomenex Luna C18 (2) column (5 mm, 150 × 4.6 mm i.d.). The mobile phase consisted of 0.05% formic acid in water (A) and acetonitrile (B) with a flow rate of 0.7 mL/min. The gradient conditions were as follows: 0–30 min, 10–100% B. The injection volume was 10 μL and the column temperature was maintained at 30°C. MS data were acquired in positive ion mode using a scan range of 150–1500 m/z. The parameters were as follows: capillary voltage of 3000 V, dry gas flow rate of 12 L/min, nebulizer pressure of 35 psig, and dry gas temperature of 350 °C. Additionally, the *n*-BuOH fraction (200 mg) was injected into a Gilson semi-preparative HPLC (acetonitrile/water with 0.05% formic acid, gradient from 10:90 to 23:77 over 160 min, flow rate of 8.0 mL/min) and further purified to obtain roseoside (0.5 mg, *t*_R_ = 44.8 min), isoquercitrin (0.2 mg, *t*_R_ = 76.6 min), umbelliferone (3.5 mg, *t*_R_ = 79.9 min) and scopoletin (0.8 mg, *t*_R_ = 85.8 min). The obtained compounds were analyzed using HPLC-MS and a Bruker 500 MHz spectrometer (Billerica, MA, USA), and the results were compared with the standard compound data.

### 4.8. Structure Prediction of HCV Protein–Roseoside Complexes

The structural data of roseoside and HCV NS5A/B replicase were obtained from the PubChem database (CID: 9930064) and the RCSB Protein Data Bank (PDB code: 1YUY), respectively. Using the AutoDock program, the data for roseoside and HCV NS5A/B replicase were converted into pdbqt format, and a grid box was set up. The optimal site where roseoside binds to HCV NS5A/B replicase was determined by calculating the x, y and z coordinates and rotational values. Using these calculated values, the optimal structure with the most stable binding energy between roseoside and HCV NS5A/B replicase was predicted using the AutoDock Vina program. The predicted roseoside-HCV NS5A/B replicase complex was visualized in 3D using the PyMOL program (version 3.0.3). By entering code, the binding sites and amino acid residues of HCV NS5A/B replicase to which roseoside binds were indicated.

### 4.9. Plasmid Construction

The HCV JFH-1 NS5B vector was constructed for the expression of histidine (His)/mannose-binding protein (MBP)-tagged active protease. NS5B fragments were obtained from JFH-1 expression plasmids (provided by T. Wakita, National Institute of Infectious Diseases and Toray Industries, Tokyo, Japan) (accession no: AB047639.1) via PCR amplification with the following primers: NS5B_F, GGGGACAAGTTTGTACAAAAAAGCAGGCTCAATGTCCATGTCATACTCCTGGACCGGGGCT; NS5B_R, GGGGACCACTTTGTACAAGAAAGCTGGGTATTACCGAGCGGGGAGTAGGAAGAGGCCTA. Amplified products were cloned into pDEST-HisMBP using the Gateway cloning system (Invitrogen, Carlsbad, CA, USA). pDEST-HisMBP was a gift from David Waugh (Addgene plasmid # 11085).

### 4.10. Protein Expression and Purification

The HCV NS5B protein with a His/MBP tag at the N-terminus was expressed in Rosetta™ 2 (DE3) Singles™ Competent Cells (Novagen, Madison, WI, USA) [40]. The recombinant protein expression vector was transformed into DE3 cells and grown in Luria–Bertani (LB) medium with selected antibiotics (100 μg/mL ampicillin and 25 μg/mL chloramphenicol). A single colony was inoculated in identical medium until the OD_600_ value reached 0.5–0.6. Protein expression was subsequently induced by culturing in medium containing 0.2% glucose and 0.2 mM isopropyl b-D-1-thiogalactopyranoside (IPTG) for 18 h at 18 °C [41]. Cells were harvested via centrifugation at 1000× *g* for 10 min and lysed by sonication in lysis buffer (1.25% Triton X-100, 50 mM Tris-HCl, 150 mM NaCl, 2 mM EDTA, 1 mg/mL lysozyme; pH 7.4). Lysates were centrifuged at 15,000× *g* for 10 min and the supernatant was subjected to His-trap purification. His/MBP tag-fused HCV NS3-NS4A was purified using HisPur™ Ni-NTA Spin Columns (Thermo Scientific, Waltham, MA, USA) as described by the manufacturer. The eluted protein fraction was stored in aliquots at −80 °C.

### 4.11. NS5A/B Replicase Assay

The RdRp activity of HCV NS5A/B was determined fluorometrically using SYTO RNASelect Green Fluorescent cell Stain (Invitrogen, S32703), which binds dsRNA but not ssRNA templates. RNA polymerization was performed using 50 mM Tris-HCl, 2.5 mM MnCl_2_, 500 μM ATP, 20 μg/mL poly-U, 0.1 mg/mL BSA, 0.5 μM SYTO RNASelect and 25 nM HCV JFH-1 strain NS5B [42]. NS5B proteins were treated with DMSO, dasabuvir or roseoside from the *n*-BuOH fraction of the KKE for 15 min at room temperature and the replicase assay was performed. At 1 h post-reaction, fluorescence was measured with the GloMax^®^ Discover System (Promega) (excitation/emission: 475 nm/500–550 nm). Relative fluorescence units were calculated by removal of the background fluorescence (Mock) from each unit.

## 5. Conclusions

The KKE induced a significant reduction in HCV RNA levels, and the activity-guided fractionation of the KKE revealed that the *n*-BuOH fraction was the most effective, without inducing cytotoxicity. The HPLC-MS analysis identified the presence of roseoside in the *n*-butanol fraction of the KKE, which exhibited antiviral activity against HCV. Roseoside inhibited HCV RNA replication by targeting the viral NS5A/B replicase without exerting cytotoxicity.

## Data Availability

The data of the present study are included in the manuscript. The raw data of the present study are available from the corresponding author upon reasonable request.
